# Behavioral Mechanisms of a Digital Health Intervention for Self-Management in Type 2 Diabetes Mellitus: Prospective Longitudinal Cohort Study

**DOI:** 10.2196/79081

**Published:** 2026-08-10

**Authors:** Yibo Wu, Yang Ni, Yang Jiang, Zijie Xu, Hewei Min, Ping Chen, Xinbao Gu, Bingyang Kong, Yadi Gan, Pei Li, Mingzi Li, Xiaohui Guo, Xuxi Zhang, Aijuan Ma, Xinying Sun

**Affiliations:** 1 School of Public Health, Peking University, 38 Xueyuan Road, Haidian District, Beijing, 100191, China, 86 010-82801620; 2 Wuhu Coach Hospital, Wuhu, China; 3 North China University of Science and Technology, Tangshan, China; 4 City University of Hong Kong, Hongkong, China; 5 Capital Medical University, Beijing, China; 6 Chinese Center for Disease Control and Prevention, Beijing, China; 7 PopMed Technology Incorporate, Beijing, China; 8 School of Nursing, Peking University, Beijing, China; 9 Department of Endocrinology, Peking University First Hospital, Beijing, China; 10 Department of Social Medicine and Health Education, School of Public Health, Peking University, Beijing, China

**Keywords:** artificial intelligence, self-management, self care, type 2 diabetes mellitus, structural equation modeling

## Abstract

**Background:**

Sustaining self-management is critical for optimizing clinical outcomes in individuals with type 2 diabetes mellitus (T2DM). Although digital health interventions (DHIs) have shown benefits for glycemic control and self-care, much of this evidence has focused on efficacy, and the behavioral mechanisms through which DHIs produce sustained effects remain insufficiently understood. Clarifying these mechanisms could inform the development of theory-driven interventions.

**Objective:**

This study examined the longitudinal behavioral pathways through which Artificial Intelligence-based Health Education Accurately Linking System, a WeChat (Tencent)–based digital health program, influences T2DM self-management, using the Extended Multi-Theory Model (MTM) of health behavior change.

**Methods:**

An explanatory sequential mixed methods prospective longitudinal cohort study was conducted among adults with T2DM (aged ≥18 y and proficient in WeChat use), recruited from 45 primary health care institutions in Beijing, China, between July 2023 and July 2024. Self-management behavior was assessed as the primary outcome using the Summary of Diabetes Self-Care Activities, and psychosocial determinants using the Extended MTM Scale and the Diabetes-related Skills Scale. Exploratory and confirmatory factor analyses assessed the construct validity of the Extended MTM Scale. Structural equation modeling examined longitudinal pathways among Artificial Intelligence-based Health Education Accurately Linking System users across baseline and 3, 6, and 12 months. For the qualitative phase, a purposive subsample was selected through maximum variation sampling based on baseline glycated hemoglobin; interviews were analyzed thematically until thematic saturation, and integrated with quantitative findings using a joint display.

**Results:**

Of the 406 enrolled participants, 391 completed baseline assessments. The Extended MTM Scale demonstrated a 6-factor, 22-item structure with excellent internal consistency (Cronbach α=0.928) and satisfactory construct validity. The structural equation modeling showed satisfactory fit (CFI=0.984, RMSEA=0.036). Changes in the social environment (*β*=0.23, 95% CI 0.07‐0.38; *P*=.003) and physical environment (*β*=0.25, 95% CI 0.10‐0.40; *P*=.001) at baseline, and diabetes-related skills at month 6 (*β*=0.16, 95% CI 0.03‐0.29; *P*=.01), were directly associated with self-management behavior at month 12, whereas behavioral confidence and emotional transformation showed no significant direct effects. Social environment changes were indirectly associated with behavioral confidence through participatory dialogue at month 3 (*β*=0.20, 95% CI 0.04‐0.36; *P*=.01; *β*=0.66, 95% CI 0.57‐0.74; *P*<.001). Thematic analysis of 17 interviews identified 3 domains: environmental context, cognitive processes, and attitudes and skills. Environmental factors converged across both data strands, while qualitative data expanded on the cognitive and attitudinal processes underlying sustained self-management.

**Conclusions:**

This study is among the first to apply the Extended MTM framework to DHI-supported T2DM self-management, with environmental factors emerging as key drivers alongside selected cognitive, attitudinal, and skills-related processes. Complementing efficacy-focused research, it illuminates the psychosocial pathways underlying sustained self-management and refines the Extended MTM in digital health contexts. These insights can inform the design of theory-driven DHIs in primary care.

## Introduction

### Background

The prevalence of type 2 diabetes mellitus (T2DM) continues to rise and has emerged as a major global public health challenge [1]. This chronic condition not only imposes a substantial burden on individual health, marked by increased risk of disability, reduced quality of life, and shortened life expectancy [2], but also results in significant medical and socioeconomic costs [3]. According to the International Diabetes Federation, China has the highest number of people with diabetes and diabetes-related deaths worldwide, and the age of onset is trending younger [4].

As a lifelong condition, effective management of T2DM largely depends on patients’ self-management capabilities [5], which encompass dietary regulation, regular physical activity, medication adherence, blood glucose monitoring, foot care, and routine eye examinations. However, awareness, treatment, and glycemic control rates in China remain suboptimal [6], highlighting a substantial gap in diabetes self-management practices. Therefore, the implementation of professionalized, standardized, and evidence-based self-management strategies is critically important for individuals living with T2DM.

In recent years, digital health interventions (DHIs) have shown great promise in supporting diabetes self-management [7,8]. By leveraging smartphones and other mobile devices, DHIs can overcome the time and space limitations of traditional community-based care, promote behavioral changes, and enhance the accessibility and equity of health services. To this end, our research team developed the Artificial Intelligence-based Health Education Accurately Linking System (AI-HEALS), implemented on the WeChat (Tencent) platform under the name Peking Diabetes Butler. This mobile application integrates knowledge graphs and natural language processing technologies and comprises an AI chatbot, tailored health education delivery, and blood glucose monitoring reminders [9]. It aims to provide patients with personalized and continuous support for diabetes self-management.

### Theoretical Framework

Various theoretical models have been used in diabetes self-management interventions, including the Health Belief Model, Social Cognitive Theory, Self-Efficacy Theory, and social support frameworks [10]. These models emphasize strengthening individuals’ cognitive, psychological, and social resources to address barriers to behavior change. However, most conventional models primarily focus on behavior initiation and provide more limited insight into the processes that support long-term maintenance. The Multi-Theory Model (MTM) of health behavior change has emerged as an integrative framework that draws on key elements from several traditional theories. MTM is particularly relevant to diabetes self-management, which involves multiple sustained behaviors such as dietary regulation, physical activity, blood glucose monitoring, and medication adherence. The model conceptualizes behavior change in 2 phases: behavioral initiation, including participatory dialogue, behavioral confidence, and changes in the physical environment, and behavioral sustenance, including emotional transformation, practice for change, and changes in the social environment [11,12].

To date, MTM has been applied in interventions targeting smoking cessation [13], dietary behavior [14], and physical activity [15], with evidence of benefit for quality of life and disease-related psychological outcomes [16,17]. However, most applications have been conducted in offline settings, and empirical evidence supporting MTM in DHIs remains limited. In addition, previous studies suggest that the explanatory performance of MTM varies across behavioral contexts and populations [11], indicating a need to examine its applicability in specific settings. In diabetes self-management, sustained behavior depends not only on motivation and environmental support but also on patients’ ability to carry out routine self-care tasks, such as blood glucose monitoring, dietary adjustment, and medication management [18]. Therefore, the present study used MTM as the core theoretical framework and incorporated diabetes-related skills as an additional construct to better capture the practical demands of diabetes self-management in a digital intervention context.

### Study Objective

Therefore, this mixed methods study aimed to examine the behavioral mechanisms through which AI-HEALS influences diabetes self-management using an Extended MTM framework and to integrate quantitative and qualitative evidence regarding these mechanisms.

## Methods

### Study Design

This study was a mixed methods prospective longitudinal cohort substudy within the AI-HEALS program (Chinese Clinical Trial Registry: ChiCTR2300068952; registered March 2023), examining the behavioral mechanisms underlying diabetes self-management. An explanatory sequential mixed methods design was used, in which data were collected prospectively. Reporting followed the Good Reporting of A Mixed Methods Study guideline [19]. The completed checklist is provided in Checklist 1.

The quantitative phase comprised 2 components. First, the psychometric properties and construct validity of the Extended MTM Scale were assessed using baseline data through exploratory and confirmatory factor analyses. Second, to examine the hypothesized behavioral pathways, AI-HEALS users were followed over 12 months, with self-report assessments at 4 time points (baseline and 3, 6, and 12 months), and structural equation modeling was applied to these repeated measures. In the subsequent qualitative phase, a purposive subsample of the AI-HEALS users was interviewed to interpret and contextualize the quantitative findings.

**Figure 1.**
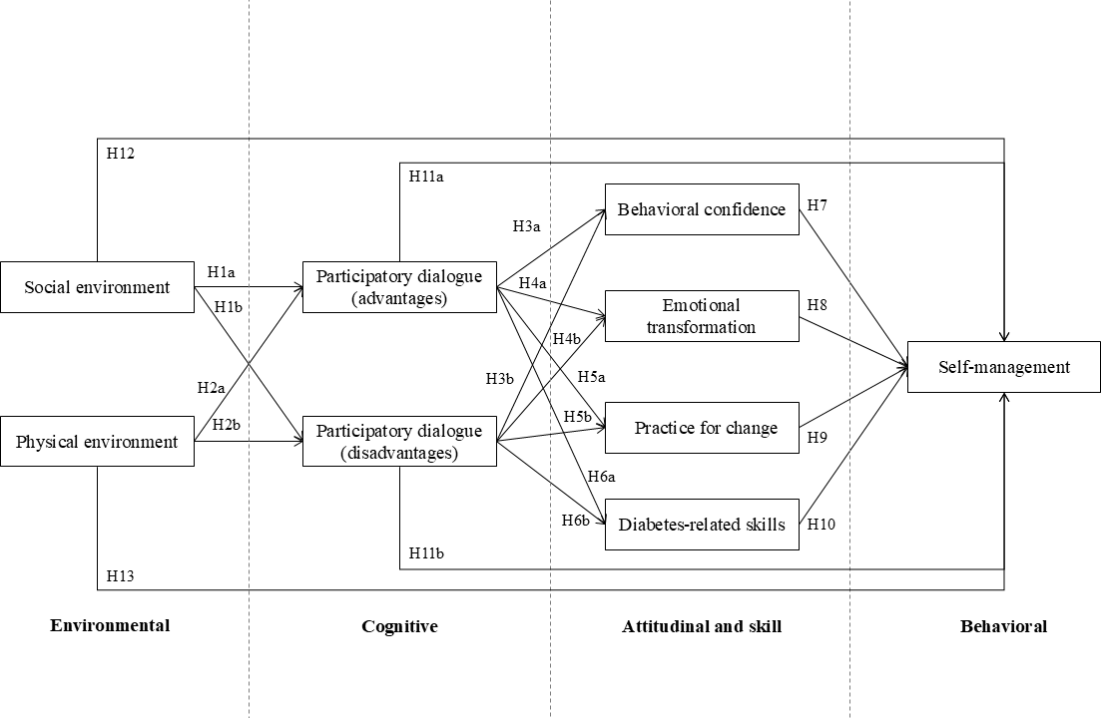
Hypothesized model of the extended Multi-Theory Model of health behavior change for diabetes self-management behavior. The hypothesized model comprises 13 pathways (H1–H13) linking environmental, cognitive, attitudinal and skill, and behavioral domains among adults with type 2 diabetes mellitus. H1–H2 represent pathways from environmental factors to participatory dialogue; H3–H6 represent pathways from participatory dialogue to attitudinal and skill-related constructs; H7–H10 represent pathways from attitudinal and skill-related constructs to self-management behavior; H11–H13 represent hypothesized direct pathways from environmental and cognitive constructs to self-management behavior.

The Extended MTM framework was organized into 4 domains—environmental, cognitive, attitudinal and skills, and behavioral—reflecting the hypothesized progression from contextual conditions to proximal individual-level processes and, ultimately, to self-management behavior. Figure 1 presents the hypothesized model, comprising 13 hypothesized pathways (H1–H13) linking environmental factors to cognitive processes, cognitive processes to attitudinal and skill-related constructs, and all preceding domains to self-management behavior. This framework guided both the quantitative path analysis and the qualitative inquiry.

### Intervention

Participants received standard diabetes primary care supplemented by AI-HEALS, a personalized DHI delivered via WeChat. AI-HEALS comprised an interactive AI chatbot, tailored health education delivery, and individualized blood glucose monitoring reminders. During the first 3 months, participants received at least one tailored self-management message per day and were provided with an operational guide in the first week. After the initial 3 months, the program shifted from proactive daily message delivery to a maintenance phase in which participants retained access to the chatbot, educational resources, and reminder functions.

### Setting and Participants

A total of 406 adults with T2DM were enrolled from 45 primary health care institutions in Daxing and Shunyi districts of Beijing. Eligibility criteria included age≥18 years, diagnosis of T2DM, residency in Beijing with less than one month of annual travel outside the city, smartphone ownership and proficiency in WeChat use, no use of psychiatric medications, and the ability to independently complete questionnaires.

Baseline data from participants with complete assessments were randomly split into 2 subsets: one for item selection via exploratory factor analysis (EFA), and the other for scale validation via confirmatory factor analysis (CFA). Longitudinal structural equation modeling (SEM) was conducted among participants who used AI-HEALS, examined across baseline, 3, 6, and 12 months.

For the interviews, participants were purposively sampled from among AI-HEALS users who had completed the quantitative assessments. A maximum variation sampling strategy based on glycated hemoglobin (HbA_1c_) levels measured at baseline was applied to capture diverse diabetes management profiles. Recruitment continued until data sufficiency was achieved across the range of sampling variation.

### Quantitative Data Collection

Quantitative data were collected using coded questionnaires between July 2023 and July 2024. At baseline, all eligible participants completed the Extended MTM scale for construct validity assessment. Factor analyses were conducted to assess the construct validity of the Extended MTM Scale. Participants who used AI-HEALS completed follow-up assessments at 3, 6, and 12 months.

### Qualitative Data Collection

Qualitative data were collected through one-on-one telephone interviews conducted by the author at the 6-month follow-up (January 8 and February 20, 2024). After obtaining verbal informed consent, interviews were conducted privately with only the interviewer and participant present. Interviews continued until thematic saturation was reached, defined as the point at which no new themes or codes emerged from successive interviews. A semistructured interview guide was used to explore participants’ experiences with AI-HEALS, covering usage behaviors, perceived usefulness and accessibility, perceived impact on self-management (including diet, physical activity, blood glucose monitoring, and medication adherence), and suggestions for improvement. The full interview guide is provided in Multimedia Appendix 1.

### Ethical Considerations

The study was approved by the Peking University Biomedical Ethics Committee (IRB00001052-22058). Informed consent was obtained from all participants prior to participation in any component of the study. Written consent was obtained before the completion of the quantitative surveys, and verbal consent was obtained prior to the telephone interviews. Prior to analysis, all data were deidentified by replacing personal identifiers with coded study identifiers, with the linkage key stored separately; no images or materials in the manuscript or supplementary files contain information that could identify individual participants. Participants who completed all follow-up assessments in the quantitative phase received a gift valued at approximately 40 Chinese Yuan (CNY; approximately US $5‐6 [1]), and those who participated in the qualitative interviews received a gift valued at approximately 100 CNY (approximately US $13‐14) upon completion.

### Measurements

Baseline data included sociodemographic and behavioral variables (eg, sex, age, education, marital and employment status, household income, smoking, and alcohol consumption). The following validated self-report instruments were administered: (1) Chinese Version of the Extended MTM Scale (Developed specifically for this study based on the MTM [12]), this 5-point Likert scale (1=strongly disagree, 5=strongly agree) assesses cognitive and behavioral constructs associated with diabetes self-management. Scale development and psychometric evaluation, including reliability and validity testing, are described in the Results section. (2) Diabetes-related Skills Scale: This 12-item scale evaluates patients’ competencies in dietary planning, physical activity adjustment, blood glucose monitoring, and hypoglycemia prevention [20]. Responses are scored on a 5-point scale, with higher scores indicating better skills. In the current study, the scale showed excellent internal consistency (Cronbach α=0.912). (3) Summary of Diabetes Self-Care Activities: This 10-item instrument assesses the frequency of diabetes self-management behaviors over the past week, across 5 domains: general diet, specific diet, exercise, blood glucose testing, and foot care [21]. Responses range from 0 to 7 days, with total scores from 0 to 77; higher scores reflect better self-care. Internal consistency in this sample was acceptable (Cronbach α=0.736).

### Statistical Analyses

To validate the Extended MTM scale, baseline data from participants with complete assessments were randomly split into 2 subsets for item screening and confirmatory evaluation. Item selection was based on EFA, item-total correlations, and internal consistency (Cronbach α). Construct validity was further assessed using CFA.

Sample size for the path analysis was determined based on empirically derived SEM criteria, including a ratio of approximately 10‐20 observations per estimated parameter and a minimum sample size of 150‐200 [22,23]. The Extended MTM model included 9‐12 free parameters in the structural component; therefore, a minimum sample size of approximately 180 was required. Path analysis using maximum likelihood estimation was conducted on longitudinal data from AI-HEALS users to estimate direct and indirect effects among MTM constructs, diabetes-related skills, and self-management behavior. Little Missing Completely at Random (MCAR) test was used to examine the missing data mechanism, and missing data were handled using full information maximum likelihood estimation. Pearson correlation coefficients were calculated to examine associations between variables. Model fit was evaluated using standard indices, including the comparative fit index (CFI), Tucker–Lewis index (TLI), and root mean square error of approximation (RMSEA), with acceptable thresholds defined as CFI and TLI>0.90 and RMSEA<0.08. Statistical significance was set at *P*<.05. All analyses were performed using IBM SPSS Statistics 27.0 (IBM Corp), IBM SPSS Amos 23.0 (IBM Corp), and Mplus 8.3 (Muthén & Muthén).

Qualitative data were analyzed using thematic analysis following the approach described by Braun and Clarke [24], adopting a primarily inductive coding strategy within a framework-informed analytic approach. Audio recordings were transcribed verbatim within 24 hours of each interview, with nonverbal cues (eg, tone, pauses, laughter) noted to preserve contextual meaning. Where local dialect terms were encountered, clarification was sought from native speakers to ensure accurate interpretation. A large language model (Doubao, version 7.3.0) was used solely for minor grammatical refinement of transcripts without altering meaning. Two researchers (YJ and BK) independently verified all transcripts against the recordings for accuracy. Transcripts were imported into NVivo 12.0 (QSR International) for analysis. Researchers first familiarized themselves with the data through repeated reading. Initial codes were generated inductively and recorded in a coding log to ensure auditability and traceability of analytic decisions. The coding log was iteratively consolidated into subthemes and higher-order themes through constant comparison within and across cases, with regular team discussions held to resolve discrepancies and reach consensus. The Extended MTM domains were used as an interpretive lens to organize emergent themes and facilitate integration with quantitative findings.

Integration of quantitative and qualitative findings was conducted using a joint display approach, mapping MTM constructs to qualitative themes to examine areas of confirmation and expansion [25,26].

## Results

### Participant Characteristics

Of 406 enrolled participants, 15 were lost to follow-up over 12 months (attrition rate 3.7%), resulting in 391 participants with complete baseline data. These baseline data were randomly split for item selection via exploratory factor analysis (EFA; n=202) and scale validation via confirmatory factor analysis (CFA; n=189). The longitudinal structural equation model was subsequently conducted among the 202 AI-HEALS users across baseline and 3, 6, and 12 months. The mean age was 56.6 (SD 11.2) years, 207/391 (53%) participants were female (Table 1), and the mean HbA_1c_ was 7.29% (SD 1.61%).

**Table 1.** Demographics and baseline characteristics of study participants^a^ (N=202). Data collected at baseline (July 2023). .

Variables	Values
Sex, n (%)	
Male	95 (47)
Female	107 (53)
Age (years), mean (SD)	56.6 (11.2)
HbA_1c_,^b^ (%), mean (SD)	7.29 (1.61)
Disease duration (years), n (%)	
≤5	57 (28.2)
6‐10	70 (34.7)
11‐20	58 (28.7)
≥21	17 (8.4)
Diabetes management, n (%)	
Diet	11 (5.4)
OADs^c^	142 (70.3)
Injectable insulin	4 (2)
OADs + injectable insulin	45 (22.3)
BMI, kg/m², n (%)	
Underweight (<18.5)	1 (0.5)
Normal weight (18.5‐23.9)	60 (29.7)
Overweight (24.0‐27.9)	75 (37.1)
Obese (≥28.0)	66 (32.7)
Waist (cm), n (%)	
Normalcy (<80)	11 (5.4)
Exceeding standard (80-94)	96 (47.5)
Obese (>94)	95 (47)
Per capita monthly income, CNY^d^^,^^e^, n (%)	
<3000	58 (28.7)
3000‐9000	114 (56.4)
>9000	30 (14.9)
Other diseases, n (%)	
0	44 (21.8)
1	49 (24.3)
≥2	109 (54)
Family history of diabetes, n (%)	
None	93 (46)
Yes	109 (54)
Smoking status, n (%)	
Non-smoker	139 (68.3)
Former smoker (quit)	10 (5)
Current smoker	54 (26.7)
Alcohol consumption status, n (%)	
Non-drinker	117 (57.9)
Former drinker (quit)	18 (8.9)
Current drinker	67 (33.1)

a Characteristics pertain to participants with type 2 diabetes mellitus (T2DM) who used Artificial Intelligence-based Health Education Accurately Linking System (AI-HEALS), recruited from community health centers and outpatient clinics in Beijing, China.

b HbA_1c_: glycated hemoglobin.

c OAD: oral antidiabetic drug.

d CNY=Chinese Yuan.

e Monthly income ≤3000 Chinese Yuan (CNY) (approximately ≤ US $400); 3000‐9000 CNY (approximately US $400‐1200); ≥9000 CNY (approximately ≥ US $1200)

For the qualitative phase, thematic saturation was achieved after 17 interviews, with no new themes emerging in the final interviews. Among them, 10 of 17 (58.8%) were women and 7 of 17 (41.2%) were men. Ten of 17 (58.8%) were aged <60 years and 7 of 17 (41.2%) were aged ≥60 years. HbA1c was <7% in 8 of 17 (47.1%) and ≥7% in 9 of 17 (52.9%). Monthly income ranged from ¥3000-¥9000 (approximately US $400‐1,200) for 10 of 17 (58.8%) participants, and 10 of 17 (58.8%) had completed high school or vocational education. Interview duration ranged from 12‐45 minutes.

### Scale Development and Validation of the Extended MTM Scale

Baseline data were randomly split for item selection via EFA and scale validation via CFA. All items demonstrated satisfactory psychometric properties, with coefficient of variation values exceeding 0.3 and item-total correlation coefficients above 0.4. EFA with varimax rotation was conducted based on the original 7-dimension MTM framework. As practice for change and emotional transformation loaded onto the same factor, expert consultation and theoretical review were undertaken, resulting in the exclusion of practice for change due to conceptual overlap with emotional transformation. The final 6-factor structure encompassed changes in the physical environment, participatory dialogue (advantages and disadvantages), behavioral confidence, emotional transformation, and changes in the social environment, with a cumulative variance explained of 86.34%, well above the recommended 50% threshold. The retained 22-item scale demonstrated excellent internal consistency (Cronbach α=0.928; greatest lower bound =0.967). CFA confirmed satisfactory structural validity (CFI=0.979, RMSEA=0.052; Multimedia Appendix 2). It should be noted that the exclusion of practice for change limited the availability of direct quantitative evidence for this dimension.

### Quantitative Findings

Descriptive statistics and correlations among Extended MTM constructs, diabetes-related skills, and self-management behaviors are summarized in Multimedia Appendix 2. The Extended MTM framework includes 4 levels: environmental (changes in the physical and social environment), cognitive (participatory dialogue advantages and disadvantages), attitudinal and skill (behavioral confidence, emotional transformation, diabetes-related skills), and behavioral (self-management). At baseline, the mean scores for changes in the physical and social environment were 9.64 (SD 3.57) and 12.35 (SD 3.03), respectively. At month 6, emotional transformation averaged 12.27 (SD 2.91), diabetes-related skills 37.66 (SD 10.03), and behavioral confidence 12.75 (SD 2.64). Self-management behaviors at month 12 had a mean score of 49.70 (SD 17.16). Emotional transformation showed strong correlation with diabetes-related skills (*r*=0.744; *P*=.008) and moderate correlation with behavioral confidence (*r*=0.474; *P*=.005). Behavioral confidence was also positively correlated with diabetes-related skills (*r*=0.471; *P*=.004).

**Figure 2.**
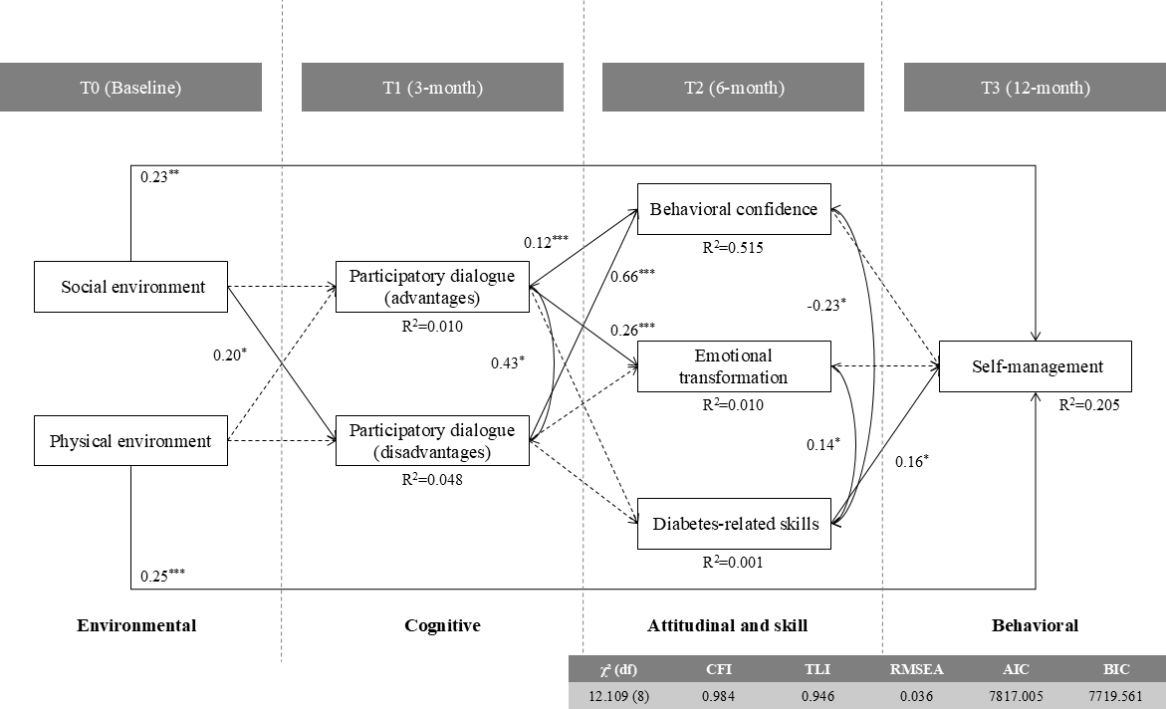
Longitudinal structural equation model of diabetes self-management behavior based on the extended Multi-Theory Model (MTM) of health behavior change. The model was estimated among adults with type 2 diabetes mellitus who used AI-HEALS (n=202), recruited from community health centers and outpatient clinics in Beijing, China, across 4 time points: baseline (T0), month 3 (T1), month 6 (T2), and month 12 (T3). Standardized path coefficients (*β*) are shown. Solid lines indicate significant paths; dashed lines indicate non-significant paths. R² values represent explained variance. *P<.05, **P<.01, ***P<.001. AIC: Akaike information criterion; AI-HEALS: Artificial Intelligence-based Health Education Accurately Linking System; BIC: Bayesian information criterion CFI: comparative fit index; MTM: Multi-Theory Model; RMSEA: root mean square error of approximation; TLI: Tucker–Lewis index;

Among the 202 AI-HEALS users included in the SEM, complete data were available for 173 (85.6%) at month 12. Little MCAR test indicated that the data were missing completely at random (*χ*²_272_=270.54; *P*=.51); missing data were therefore handled using full information maximum likelihood estimation. As shown in Figure 2, changes in the social environment (*β*=0.23, 95% CI 0.07‐0.38; *P*=.003) and physical environment (*β*=0.25, 95% CI 0.10‐0.40; *P*=.001) at baseline, and diabetes-related skills at month 6 (*β*=0.16, 95% CI 0.03‐0.29; *P*=.01) were directly associated with self-management behaviors at month 12, supporting H12, H13, and H10. In contrast, behavioral confidence and emotional transformation showed no significant direct effects on self-management behaviors, leaving H7 and H8 unsupported. Changes in the social environment at baseline were significantly associated with participatory dialogue (disadvantages) at Month 3 (*β*=0.20, 95% CI 0.04‐0.36; *P*=.01; H1b), and participatory dialogue (disadvantages) was in turn significantly associated with behavioral confidence at month 6 (*β*=0.66, 95% CI 0.57‐0.74; *P*<.001; H3b). As participatory dialogue (disadvantages) was reverse-coded, these positive coefficients reflect negative underlying effects. Participatory dialogue (advantages) at month 3 was additionally associated with behavioral confidence (*β*=0.12, 95% CI 0.02‐0.23; *P*=.02; H3a) and emotional transformation (*β*=0.26, 95% CI 0.11‐0.40; *P*=.001; H4a) at month 6. All remaining hypothesized pathways were non-significant (Multimedia Appendix 2).

The model demonstrated satisfactory fit (*χ*²_8_=12.109, CFI=0.984, TLI=0.946, RMSEA=0.036), supporting the hypothesized structure of the Extended MTM framework.

### Qualitative Findings

Based on the Extended MTM, qualitative findings were organized into 3 hierarchical domains: environmental, cognitive, and attitudinal and skills, encompassing 7 themes and 18 subthemes (Multimedia Appendix 1). Below is a concise summary of each domain.

#### Environmental Domain

Changes in both physical and social environments influenced participants’ engagement in diabetes self-management. At the level of the physical environment, accessibility and usability of digital tools were key determinants of engagement. Participants were more likely to apply health information when content was perceived as relevant, clear, and easy to use. For example, one participant described using dietary information to guide daily decisions:

*If I want to eat something, I’ll check whether it’s suitable for people with diabetes. If the sugar content is high, I won’t buy it.*

[T-BJ-360, female, 62 years]

However, limitations in information reliability and clarity were also reported. Some participants expressed uncertainty about the trustworthiness of certain content, which could reduce confidence in using the platform. As one participant noted,

*Some medication info seems unreliable.*

[T-BJ-033, male, 58 years]

This suggests that inconsistencies or lack of transparency in information presentation may hinder sustained engagement.

At the level of the social environment, family involvement and broader social support played a reinforcing role. Emotional and practical support from family members enhanced motivation and facilitated adherence to self-management behaviors. For instance, one participant stated,

*My daughter and son both really care about this*.

[T-BJ-194, female, 72 years]

Participants also highlighted the importance of wider societal support for health promotion:

*I truly hope the government can promote health knowledge among young people.*

[T-BJ-194, female, 72 years]

#### Cognitive Domain

Interactions with the digital platform, together with input from health care providers and social networks, supported participants’ understanding and decision-making processes. Participants described how exposure to structured information helped them reassess prior beliefs and make more informed choices. For example, one participant noted,

*I’ve read a lot of the content. I used to think certain foods were low in sugar or sugar-free, but now I know they’re not*.

[T-BJ-182, female, 51 years]

This reflects a process of cognitive correction and improved health literacy.

In addition, the combination of digital information and offline guidance appeared to enhance perceived usefulness and credibility. As one participant stated,

*This content, combined with the community doctor’s talks, fully meets my needs.*

[T-BJ-173, female, 67 years]

Another participant emphasized the practical value of the platform:

*I’ve learned so many things through this. I’m sure it will help me solve a lot of problems going forward.*

[T-BJ-035, male, 61 years]

#### Attitudinal and Skills Domain

This domain encompassed behavioral confidence, emotional transformation, practice for change, and diabetes-related skills. Behavioral confidence emerged as a central factor influencing proactive engagement. Participants who perceived themselves as capable of managing their condition were more likely to adopt and maintain behavioral changes. For example, one participant stated,

*If you want to live long and live well, you must have confidence.*

[T-BJ-194, female, 72 years]

Trust in the platform also contributed to this confidence, as reflected in the following statement:

*The materials you provided feel really trustworthy…*

[T-BJ-407, male, 49 years]

Emotional transformation was another important process, whereby participants shifted from anxiety or passive coping to more proactive self-management. Structured information and repeated engagement appeared to support this transition. As one participant noted,

*There’s no use being anxious. Since I’ve got the disease, I might as well manage it properly...*

[T-BJ-182, female, 51]

Participants also described practice for change through reminders, self-monitoring, and the gradual incorporation of self-management behaviors into daily routines. For instance, one participant reported,

*Just last night, I forgot to check, and the Peking Diabetes Butler reminded me.*

[T-BJ-184, male, 56 years]

In parallel, participants described improvements in diabetes-related skills, particularly in blood glucose monitoring, diet, medication use, and physical activity. As one participant explained,

*My workouts are regular now... I used to exercise blindly, but now I know what I’m doing.*

[T-BJ-182, female, 51 years]

Nonetheless, some participants identified limitations in content presentation, particularly regarding clarity and format. For example, one participant suggested,

*Some of the exercise illustrations aren’t clear enough. Videos would be much better...*

[T-BJ-182, female, 51 years]

This indicates that insufficiently intuitive or engaging content may reduce usability and limit the effectiveness of skill acquisition.

Building on the subtheme analysis, we assessed whether overarching themes differed by participants’ HbA_1c_ levels. No clear differences in the overarching themes were observed across interview participants with different HbA_1c_ levels, although some variation in emphasis was noted in individual accounts.

### Integration of Quantitative and Qualitative Findings

**Figure 3.**
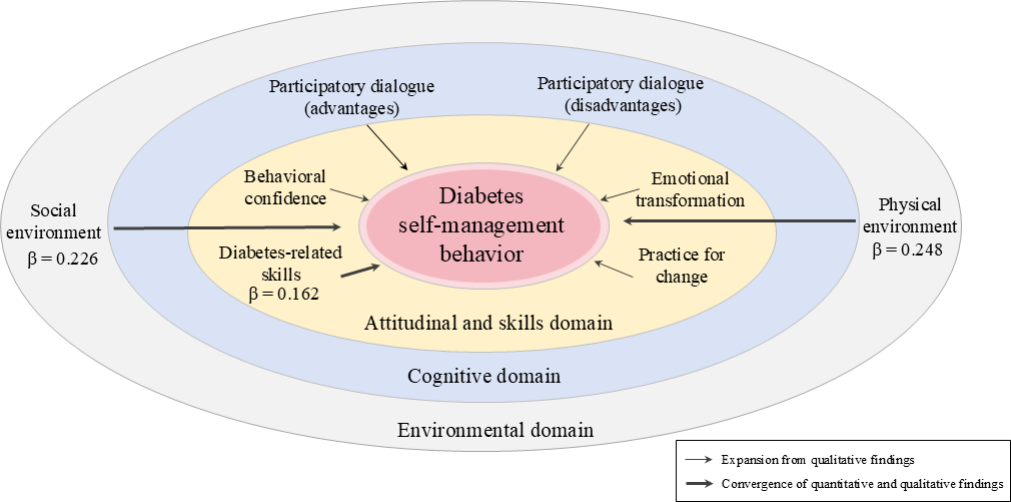
Integrated model of quantitative and qualitative findings on diabetes self-management behavior based on the extended Multi-Theory Model of health behavior change. Based on an explanatory sequential mixed methods study among adults with type 2 diabetes mellitus who used Artificial Intelligence-based Health Education Accurately Linking System, recruited in Beijing, China (n=202). Thin arrows indicate pathways derived from qualitative findings, whereas thick arrows indicate pathways supported by both quantitative and qualitative evidence. Standardized path coefficients (β) are shown for direct associations with diabetes self-management behavior identified in the quantitative model.

As illustrated in Figure 3 and Multimedia Appendix 3, integration of quantitative and qualitative data revealed both convergent and complementary patterns across the Extended MTM domains.

Environmental factors showed the strongest convergence. SEM confirmed direct associations of physical and social environment changes with self-management behavior at month 12 (β=0.25, 95% CI 0.10-0.40 and β=0.23, 95% CI 0.07-0.38), and qualitative accounts described how access to digital tools, self-management resources, and family and community support facilitated behavioral engagement. Diabetes-related skills similarly showed convergence, with a direct quantitative effect (*β*=0.16, 95% CI 0.03-0.29) corroborated by participants’ descriptions of applying glucose monitoring, dietary, and medication management skills in daily practice.

Qualitative findings expanded on constructs showing limited direct effects in the SEM. Behavioral confidence and emotional transformation were not significant direct predictors of self-management behavior, yet participants described both as present and influential in their self-management experience. Practice for change, which was excluded from the quantitative model during scale revision, was nonetheless frequently referenced by participants as part of their behavioral routines.

Participatory dialogue showed complementary patterns across both strands. Quantitative pathways linked social environment changes to behavioral confidence and emotional transformation via participatory dialogue at month 3 and month 6. Qualitative accounts described interactions with the platform, health care providers, and peers as sources of knowledge, motivation, and decision-making support.

## Discussion

### Principal Findings

This study used an explanatory sequential mixed methods design. The integrated findings provided partial confirmation and substantial expansion of the Extended MTM framework in the context of DHIs for diabetes self-management. SEM confirmed that physical and social environmental factors and diabetes-related skills had direct positive effects on self-management behavior, with environmental factors showing particularly prominent influence. Qualitative findings expanded these results by elucidating how participatory dialogue, behavioral confidence, emotional transformation, and practice for change operated alongside environmental support to facilitate sustained behavior change. Together, these findings underscore the prominent role of environmental factors, complemented by cognitive, attitudinal, and skills-related processes, in shaping effective self-management. This study represents the first application of the MTM framework to diabetes self-management and extends it based on the behavioral characteristics of this context.

Results from the SEM showed that changes in the physical and social environment had direct positive associations with diabetes self-management behaviors [27,28]. Environmental constructs showed clearer direct associations than the psychological constructs included in the model, whose pathways were not statistically significant. This pattern may be interpreted from a socioecological perspective, which views self-management behavior as embedded within broader physical and social environments rather than determined solely by individual-level psychological processes [29]. In this model, physical and social environmental changes were therefore positioned as antecedent contextual conditions for self-management, whereas psychological and skills-related constructs were modeled as downstream individual-level processes that develop within, and partly in response to, these conditions. In AI-HEALS, accessible information, reminders, and ongoing support may have strengthened the environmental conditions under which self-management occurs, which may help explain the prominence of environmental constructs in the final model. This suggests that DHIs may not act solely by strengthening internal determinants such as confidence or emotion-related processes, but may also increase the salience of contextual determinants, consistent with evidence that context-aware digital behavior change interventions dynamically tailor support based on real-world factors [30]. From an intervention perspective, strengthening supportive environmental conditions may therefore be an important entry point for promoting sustained behavioral engagement [31].

Diabetes-related skills were also found to have a direct positive influence on self-management behaviors, aligning with previous studies [32]. Diabetes self-management education should integrate behavior change techniques, such as goal setting and self-monitoring, with skills training to enhance patients’ practical capabilities [33]. Evidence suggests that skill internalization interventions can support behavioral persistence [34]; patients who acquire blood glucose monitoring skills early demonstrate significantly better self-management awareness than those without systematic training [35]. Neuroscientific studies suggest that skilled individuals show reduced activation in decision-related brain regions like the prefrontal cortex [36]. Skill internalization may shorten the neural pathway between cognitive appraisal and behavioral execution, thereby improving the efficiency and automatization of behavioral performance [37]. These findings suggest that practical skill acquisition may represent a more proximal determinant of behavioral outcomes than attitudinal processes alone, with implications for the design of DHIs that prioritize applied skills training alongside informational content.

This study found that changes in the social environment indirectly enhanced behavioral confidence through participatory dialogue (disadvantages), while participatory dialogue (advantages) directly improved behavioral confidence and emotional transformation. These findings align with previous research. In T2DM management, participatory strategies such as interactive education and motivational interviewing have been shown to strengthen behavioral confidence and emotional resonance, thereby improving patients’ motivation for self-management [38,39]. Encouraging a focus on positive emotions, such as hope and achievement, can reduce fear and helplessness and promote behavioral engagement [40]. Experience sharing and emotional support in online communities may further enhance self-efficacy as an extension of social support [41]. However, neither behavioral confidence nor emotional transformation demonstrated a significant direct effect on self-management behavior in the SEM, suggesting that their influence may operate indirectly through skills development and routine consolidation rather than directly driving behavioral outcomes. Prior meta-analyses have indicated that knowledge-based interventions exert limited behavioral impact in the absence of accompanying skills training [42], which may partly account for this pattern. This interpretation was further supported by qualitative accounts, in which participants described confidence and emotional regulation as enabling conditions for sustained engagement rather than immediate behavioral triggers.

Qualitative findings further suggest that improvements in diabetes self-management are shaped by the interplay between environmental accessibility, cognitive support, and the development of attitudes and practical skills. At the environmental level, the accessibility and usability of the AI-HEALS platform appear to provide a foundation for behavioral engagement, facilitating access to structured health information and ongoing support. In addition to these system-level features, participants frequently described the importance of family involvement and professional guidance in sustaining behavioral continuity, indicating that broader social support contexts may complement DHIs in reducing barriers to long-term engagement. These observations are consistent with prior evidence indicating that supportive environments can facilitate sustained self-management in patients with T2DM [31,43]. From a cognitive perspective, interaction with the platform appeared to support processes of understanding, appraisal, and decision-making. The delivery of timely and structured information, together with interactive feedback, may help reduce informational asymmetry and enhance users’ confidence in interpreting and applying health guidance. Rather than functioning as a standalone driver, this cognitive support seems to operate in conjunction with external reinforcement from health care providers and personal networks. The use of interactive digital platforms and conversational technologies has been shown to enhance user trust and engagement in health communication [44,45]. At the level of attitudes and skills, behavioral confidence and emotional adjustment emerged as closely linked processes that support sustained self-management. Participants described how increased confidence enabled them to translate concerns related to glycemic control into actionable strategies, while repeated engagement with the platform facilitated the gradual internalization of self-management skills. Over time, this process contributed to the establishment of routine practices across key domains, including glucose monitoring, dietary regulation, physical activity, and medication adherence, reflecting a transition towards more autonomous self-regulation. This interpretation is supported by prior research highlighting the role of emotional regulation and self-efficacy in sustaining behavioral change [46,47]; as well as behavior change techniques embedded in DHIs [48]. Notably, practice for change—though excluded from the quantitative model due to scale revision—was consistently described by participants as a mechanism linking knowledge acquisition to behavioral maintenance, suggesting its continued theoretical relevance within the Extended MTM framework. The absence of quantitative evidence for this dimension reflects a methodological limitation of the scale revision process rather than a theoretical dismissal of its role. Future studies should consider retaining or separately operationalizing practice for change to enable fuller quantitative testing of the Extended MTM framework.

Building on these observations, variation in participants’ accounts suggested that behavioral processes are shaped by the relative prominence of barriers and self-regulatory capacity. Participants with comparatively higher HbA_1c_ at month 6 more often emphasized constraints related to symptom burden and practical barriers, whereas those with lower HbA_1c_ levels tended to highlight established routines and greater behavioral confidence [49]. These differences illustrate how behavioral priorities and self-regulatory strategies vary across individuals, highlighting the dynamic nature of diabetes self-management processes. In this context, DHIs may benefit from adapting to these differing needs, particularly by strengthening external support and providing more tailored guidance for individuals facing greater behavioral constraints [50,51]. These observations also highlight areas for further optimization of the AI-HEALS system, as qualitative findings indicated concerns regarding the reliability of certain medication information and the clarity of visual content, alongside limited adaptability to individual needs, particularly among older adults and those with lower digital literacy. Addressing these issues may enhance system effectiveness through improved interface design, incorporation of multimodal content such as video-based guidance, and greater adaptability to diverse user needs, while closer integration with broader care systems and real-world support structures, including community-based platforms [52,53], may further support sustained engagement in diabetes self-management.

Grounded in the Extended MTM, this study demonstrates that AI-HEALS supports diabetes self-management through a pathway of environmental facilitation, cognitive reinforcement, and attitudinal and skill consolidation. Across both data strands, environmental factors emerged as the most consistently supported drivers of behavior change, while cognitive and attitudinal processes showed complementary roles that were more fully captured through qualitative inquiry. The convergence of findings across these domains supports the applicability of the Extended MTM as an explanatory framework for DHI-supported behavior change in chronic disease management.

### Limitations

There are several limitations that should be recognized. First, participants were recruited from community health centers in Beijing, which may limit the generalizability of findings to other geographic, cultural, or health care contexts. Second, the sample may have been subject to selection bias, as less cooperative or less digitally proficient patients may have been systematically excluded. Third, reliance on self-reported outcome measures may introduce recall bias, potentially affecting the accuracy of self-management behavior assessments. Fourth, although the longitudinal design and SEM allowed examination of temporal pathways, SEM identifies associations consistent with hypothesized causal structures but cannot establish causality definitively. Fifth, the findings are based on AI-HEALS, a single program delivered via WeChat, and may not generalize to other digital health programs with different technological platforms, user interfaces, or engagement strategies. Sixth, the exclusion of the practice for change dimension during scale revision precluded its quantitative examination, limiting the completeness of the Extended MTM framework testing.

### Conclusions

This study is among the first to apply the Extended MTM framework to diabetes self-management, demonstrating that environmental factors, alongside selected cognitive, attitudinal, and skills-related processes, play an important role in sustaining behavior change among AI-HEALS users. While environmental factors and diabetes-related skills showed direct associations with self-management behavior, behavioral confidence and emotional transformation appeared to operate indirectly, illuminating psychosocial mechanisms that efficacy-focused evaluations alone cannot capture. These findings contribute to the theoretical refinement of the Extended MTM in digital health contexts and suggest that prioritizing environmental support and skill-building in the design of digital health programs may help sustain patient engagement and self-management in primary care settings.

## Supplementary material

10.2196/79081 Multimedia Appendix 1 Interview material.

10.2196/79081 Multimedia Appendix 2 Supplementary quantitative data.

10.2196/79081 Multimedia Appendix 3 Joint display of quantitative and qualitative findings.

10.2196/79081 Checklist 1 GRAMMS checklist.

## Abbreviations

AI-HEALS: Artificial Intelligence-based Health Education Accurately Linking System
CFA: confirmatory factor analysis
CNY: Chinese Yuan
DHIs: digital health interventions
EFA: exploratory factor analysis
HbA_1c_: glycated hemoglobin
MCAR: Missing completely at random
MTM: Multi-Theory Model
RMSEA: root mean square error of approximation
SEM: structural equation modeling
T2DM: type 2 diabetes mellitus
TLI: Tucker–Lewis index
