# Supplementary material for: Behavioral Mechanisms of a Digital Health Intervention for Self-Management in Type 2 Diabetes Mellitus: Prospective Longitudinal Cohort Study
Source: JMIR Mhealth Uhealth. 2026 Aug 10;14:e79081. doi: 10.2196/79081 (PMC13455579; doi:10.2196/79081)
Supplement: Supplementary file 1 [file mhealth-v14-e79081-s001.docx]

**Multimedia Appendix 3. Qualitative Study Materials**

# Section 1. Semi-Structured Interview Guide

The following guide was used to explore participants' experiences with the PKU Diabetes Butler (AI-HEALS). Interviewers were encouraged to ask follow-up questions based on participants' responses. The guide was not theory-driven; topics were selected to capture a broad range of user experiences.

## Overall Impressions

• Which specific features of the program did you use?

• How frequently did you use the program?

• To what extent did the diabetes-related information meet your needs?

• How easy was it to understand and use the program's functions?

## Suggestions and Feedback

• Did you encounter any problems while using the program?

• Did the functions related to medication reminders, exercise reminders, blood glucose monitoring, and dietary recommendations operate as expected? What suggestions do you have?

• Do you find the language and terminology used in the program appropriate?

## User Experience

• At what times of day do you typically use the program?

• In what contexts or environments do you usually use the program?

• Which features do you tend to use most frequently?

• Which features have had a positive impact on your health, and which have had little or negative effects?

• (For participants with low usage frequency) What are the main reasons for low usage?

• (For participants with low usage frequency) What factors have prevented consistent use?

• (For participants with low usage frequency) If issues were addressed, would you continue using the program?

## Changes Associated with Program Use

• What specific impacts has the program had on your health, both positive and negative?

• How has the program influenced your blood glucose control and related behaviours?

• After using the program, have you become more attentive to diet and exercise?

• What diabetes-related skills have you learned through the program?

• To what extent has your understanding of diabetes improved after using the program?

• Have there been any changes in your external environment during participation, and how have these affected your self-management?

## Additional Questions

• How confident and willing are you to strengthen your self-management in the future?

• To what extent do you think the program has contributed to your diabetes management?

• Do you have any additional suggestions or comments?

Closing: The interviewer may ask follow-up questions based on participants' responses. Thank you for your time and participation.

# Section 2. Participant Characteristics

A total of 17 participants were purposively selected from among AI-HEALS users using maximum variation sampling based on baseline HbA_1c_ level (<7% vs ≥7%). Interviews were conducted between January 8 and February 20, 2024, with durations ranging from 12-45 minutes. Participant characteristics are presented in Table 1.

**Table 1.** Characteristics of qualitative study participants (N=17).

| **ID** | **Sex** | **Age** | **Household Registration** | **Monthly Income (CNY)** | **Education** | **Baseline HbA*_1c_* (%)** | **Employment Status** |
| --- | --- | --- | --- | --- | --- | --- | --- |
| T-BJ-182 | F | 51 | Agricultural | >9,000 | High School | 4.4 | Employed |
| T-BJ-035 | M | 61 | Agricultural | ≤3,000 | High School | 5.7 | Unemployed |
| T-BJ-360 | F | 62 | Non-agricultural | 3,000-9,000 | High School | 5.9 | Retired |
| T-BJ-033 | M | 58 | Agricultural | ≤3,000 | Junior High School | 6.1 | Employed |
| T-BJ-342 | M | 50 | Agricultural | 3,000-9,000 | High School | 6.5 | Employed |
| T-BJ-194 | F | 72 | Non-agricultural | 3,000-9,000 | Junior High School | 6.7 | Retired |
| T-BJ-173 | F | 67 | Non-agricultural | 3,000-9,000 | High School | 6.9 | Retired |
| T-BJ-199 | M | 51 | Non-agricultural | ≥9,000 | High School | 6.9 | Employed |
| T-BJ-148 | F | 73 | Non-agricultural | 3,000-9,000 | Associate Degree | 7.1 | Retired |
| T-BJ-264 | M | 65 | Non-agricultural | 3,000-9,000 | High School | 7.1 | Employed |
| T-BJ-062 | M | 52 | Non-agricultural | ≥9,000 | Associate Degree | 7.3 | Unemployed |
| T-BJ-326 | F | 30 | Non-agricultural | ≥9,000 | Associate Degree | 7.6 | Employed |
| T-BJ-407 | M | 49 | Non-agricultural | ≥9,000 | Bachelor | 7.6 | Employed |
| T-BJ-184 | M | 56 | Agricultural | 3,000-9,000 | Junior High School | 7.8 | Employed |
| T-BJ-327 | M | 69 | Non-agricultural | 3,000-9,000 | Secondary Specialized | 8.9 | Retired |
| T-BJ-267 | F | 54 | Agricultural | ≤3,000 | Junior High School | 10.5 | Retired |
| T-BJ-384 | M | 58 | Agricultural | ≤3,000 | Junior High School | 11.1 | Retired |

*Note:* CNY = Chinese Yuan; HbA_1c_ = glycated hemoglobin. Monthly income ≤3,000 CNY (approximately ≤ US $400); 3,000–9,000 CNY (approximately US $400–1,200); ≥9,000 CNY (approximately ≥ US $1,200).

# Section 3. Thematic Coding Framework

Interview data were analysed using thematic analysis. The extended MTM domains served as an organising framework during the analytical stage. Analysis yielded seven overarching themes and 18 subthemes. Table 2 presents the coding framework with participant engagement frequency for each subtheme.

**Table 2.** Thematic coding framework (N=17).

| **Theme** | **Subtheme** | **No. of Participants** | **Percentage** | **No. of References** |
| --- | --- | --- | --- | --- |
| Changes in Physical Environment | Accessibility and usability of self-management resources | 10 | 58.82% | 15 |
|  | Accessibility of the PKU Diabetes Butler | 13 | 76.47% | 39 |
| Changes in Social Environment | Support and supervision from family members | 11 | 64.71% | 17 |
|  | Support from peer groups and the PKU Diabetes Butler | 8 | 47.06% | 21 |
|  | Other social environmental changes | 9 | 52.94% | 26 |
| Participatory Dialogue | Two-way communication using the PKU Diabetes Butler | 15 | 88.24% | 65 |
|  | Interaction with healthcare professionals | 8 | 47.06% | 25 |
|  | Interaction with others | 9 | 52.94% | 17 |
| Behavioural Confidence | Confidence in one's own abilities and glycaemic control | 15 | 88.24% | 35 |
|  | Confidence in the PKU Diabetes Butler | 12 | 70.59% | 26 |
| Emotional Transformation | Improved emotional regulation skills | 12 | 70.59% | 18 |
|  | Transforming emotion into motivation | 9 | 52.94% | 16 |
| Practice for Change | Behavioural monitoring and reflection | 9 | 52.94% | 23 |
|  | Behavioural adjustment and maintenance | 16 | 94.12% | 32 |
| Diabetes-related Skills | Blood glucose monitoring skills | 15 | 88.24% | 44 |
|  | Dietary management skills | 15 | 88.24% | 70 |
|  | Physical activity management skills | 14 | 82.35% | 43 |
|  | Medication management skills | 12 | 70.59% | 24 |

*Note*: Number of participants = number of interview transcripts containing the subtheme; Percentage = proportion of total participants (N=17); Number of references = total coded instances across all transcripts.

# Section 4. Representative Quotes by Theme and Subtheme

Table 3 presents representative quotes for each subtheme alongside an analytic note summarising the key insight. Quotes have been translated from Mandarin Chinese and lightly edited for grammatical clarity without altering meaning.

**Table 3.** Representative quotes by theme and subtheme.

| **Theme** | **Subtheme** | **Representative Quotes** | **Analytic Note** |
| --- | --- | --- | --- |
| ***Environmental Domain*** | | | |
| Changes in Physical Environment | Accessibility and usability of self-management resources | "I bought a stationary bike to ride and exercise regularly." (T-BJ-326, F, 30) "I bought a digital scale and an electronic balance to weigh staple foods, never more than 60 grams per serving." (T-BJ-327, M, 69) | Participants actively acquired self-management tools to support behaviour change. |
|  | Accessibility of the PKU Diabetes Butler | "WeChat constantly reminds me—eat more vegetables, less rice and steamed buns, more whole grains." (T-BJ-267, F, 54) "Some medication info seems unreliable." (T-BJ-033, M, 58) | Platform use supported health literacy, though information reliability remained a concern for some. |
| Changes in Social Environment | Support and supervision from family members | "My daughter and I now monitor each other. When we're out and see something really tempting, we just nod and walk away." (T-BJ-194, F, 72) | Family involvement reinforced motivation and dietary adherence. |
|  | Support from peer groups and the PKU Diabetes Butler | "I think the PKU Diabetes Butler is really practical, I plan to recommend it to my friends." (T-BJ-199, M, 51) "I know people with diabetes, but I don't plan to recommend the PKU Diabetes Butler to them." (T-BJ-062, M, 52) | Social network influence varied across participants; some actively endorsed peer sharing while others did not. |
|  | Other social environmental changes | "Services are shifting to community level... I can get my medication and see a doctor right in the community." (T-BJ-173, F, 67) "Acarbose used to be expensive. But now that it's covered by insurance, I can get it for just a few yuan." (T-BJ-148, F, 73) | Improved community healthcare access and insurance coverage facilitated sustained self-management. |
| ***Cognitive Domain*** | | | |
| Participatory Dialogue | Two-way communication using the PKU Diabetes Butler | "I've learned so many things through this. I'm sure it will help me solve a lot of problems going forward." (T-BJ-035, M, 61) "This content, combined with the community doctor's talks, fully meets my needs." (T-BJ-173, F, 67) | Digital and offline information sources were complementary in reinforcing health knowledge. |
| Participatory Dialogue | Interaction with healthcare professionals | "I'm good friends with Dr. Wang from the community clinic. If I ever feel unwell, I talk to him right away." (T-BJ-173, F, 67) "He said people my age shouldn't walk too much... My limit should be 7,000 steps." (T-BJ-062, M, 52) | Physician guidance directly shaped participants' physical activity and lifestyle behaviours. |
|  | Interaction with others | "It scared me. After seeing my colleague's father pass away from diabetic complications, I started taking my medication and monitoring my blood sugar more carefully too." (T-BJ-062, M, 52) | Vicarious experiences prompted attitudinal shifts and greater adherence. |
| ***Attitudinal/Skills Domain*** | | | |
| Behavioural Confidence | Confidence in one's own abilities and glycaemic control | "If you want to live long and live well, you must have confidence." (T-BJ-194, F, 72) Interviewer: "How confident are you?" Participant: "One hundred percent!" (T-BJ-035, M, 61) | Self-efficacy was a central driver of sustained engagement in self-management behaviours. |
|  | Confidence in the PKU Diabetes Butler | "The materials you sent me feel really trustworthy. After all, they're from your department, so they must be authoritative." (T-BJ-407, M, 49) | Perceived credibility of the platform enhanced participants' confidence in following its guidance. |
| Emotional Transformation | Improved emotional regulation skills | "There's no use being anxious. Since I've got the disease, I might as well manage it properly." (T-BJ-182, F, 51) "I used to be anxious and depressed all the time. Not anymore." (T-BJ-035, M, 61) | Participants transitioned from anxiety to acceptance, supporting more proactive self-management. |
|  | Transforming emotion into motivation | "I'm almost 70—if I don't travel now, I might not get another chance." (T-BJ-360, F, 62) | Positive reframing of disease experience fostered sustained motivation for behaviour change. |
| Practice for Change | Behavioural monitoring and reflection | "Just last night, I forgot to check, and the PKU Diabetes Butler reminded me." (T-BJ-184, M, 56) "Now I have to fill out the form every day of every month. I log how much medication I've taken." (T-BJ-194, F, 72) | Digital reminders and self-logging facilitated habit formation and behavioural accountability. |
|  | Behavioural adjustment and maintenance | "If I feel like I've overeaten or eaten something I probably shouldn't have, I'll test about two hours later." (T-BJ-360, F, 62) "I try to increase my physical activity so I won't have to rely on higher doses of medication." (T-BJ-407, M, 49) | Participants demonstrated adaptive self-regulation based on monitoring feedback. |
| Diabetes-related Skills | Blood glucose monitoring skills | "I didn't check my blood sugar this often before. Now, I monitor it seriously." (T-BJ-342, M, 50) "I pay more attention to postprandial glucose than to fasting glucose." (T-BJ-062, M, 52) | Structured monitoring routines improved participants' awareness of glycaemic patterns. |
|  | Dietary management skills | "If a dish includes starchy items like yam or potatoes, I try to eat less rice." (T-BJ-407, M, 49) "I used to eat fruit freely. Not anymore. I rarely buy bananas or grapes." (T-BJ-173, F, 67) | Participants applied dietary knowledge to make nuanced, context-specific food decisions. |
|  | Physical activity management skills | "Now my workouts are regular, at set times... I used to work out blindly, but now I understand what scientific exercise means." (T-BJ-182, F, 51) "Some of the exercise illustrations aren't clear enough. Videos would be much better." (T-BJ-182, F, 51) | Exercise habits improved in structure and consistency, though content format limitations were noted. |
|  | Medication management skills | "I rarely forget to take my meds. I place the pillbox right on the dining table." (T-BJ-360, F, 62) "If I go out with friends and eat more than usual... do I need to increase my medication? From what I read on the Diabetes Manager, the answer is no." (T-BJ-407, M, 49) | Medication adherence was reinforced by practical strategies and platform guidance on dose management. |

*Note*: M = Male; F = Female. Participant identifiers follow the format T-BJ-[ID number]. Age reported in years.
