# Supplementary material for: Behavioral Mechanisms of a Digital Health Intervention for Self-Management in Type 2 Diabetes Mellitus: Prospective Longitudinal Cohort Study
Source: JMIR Mhealth Uhealth. 2026 Aug 10;14:e79081. doi: 10.2196/79081 (PMC13455579; doi:10.2196/79081)
Supplement: Supplementary file 2 [file mhealth-v14-e79081-s002.docx]

**Multimedia Appendix 3**

**Table S1.** Specific entries in the Multi-Theory Model (MTM) of health behavior change scale pool.

| Dimension | NO. | Item |
| --- | --- | --- |
| Changes in the physical environment | PE1 | How confident are you in your ability to engage in daily health management |
|  | PE2 | How confident are you in using an AI-based programme to support your daily health management |
|  | PE3 | How confident are you in your ability to acquire knowledge related to health management daily |
| Participatory dialogue (advantages) | AD1 | If you managed your health every day, you would become healthier |
|  | AD2 | If you managed your health every day, your fasting blood glucose would remain below 7 mmol/L |
|  | AD3 | If you managed your health every day, your diet would contain less oil, salt, and sugar |
|  | AD4 | If you managed your health every day, your daily physical activity would increase by at least 30 minutes |
|  | AD5 | If you managed your health every day, your confidence in disease management would increase significantly |
| Participatory dialogue (disadvantages) | DIS1 | If you managed your health every day, it would be very inconvenient |
|  | DIS 2 | If you managed your health every day, it would be a waste of time and energy |
|  | DIS 3 | If you managed your health every day, it would reduce your opportunities for social interaction |
|  | DIS4 | If you managed your health every day, it might cause undesirable side effects |
|  | DIS5 | If you managed your health every day, you might feel anxious, depressed, or stressed |
| Behavioral confidence | BC1 | Even if it feels troublesome, I believe I can start managing my health every day. |
|  | BC2 | Even if I feel nervous or anxious, I believe I can begin daily health management |
|  | BC3 | Even if it seems difficult, I believe I can initiate daily health management |
| Emotional transformation | ET1 | How confident are you in your ability to motivate yourself to manage your health every day |
|  | ET2 | How confident are you in using an app to monitor your daily health management |
|  | ET3 | How confident are you in your ability to overcome self-doubt regarding daily health management |
| Changes in the social environment | SE1 | How confident are you in receiving support from your family to manage your health |
|  | SE2 | How confident are you in receiving support from your friends to manage your health |
|  | SE3 | How confident are you in receiving support from healthcare professionals to manage your health |
| Practice for change | PC1 | How confident are you in using an app to monitor your daily health management |
|  | PC2 | If you face obstacles, how confident are you in maintaining your daily health management routine |
|  | PC3 | If you encounter difficulties, how confident are you in adjusting your daily health management goals accordingly |

**Table S2.** Multi-Theory Model (MTM) of health behavior change scale entries screening results (*n*=202).

| Item | Number of Ratings | | | | | CV | Cronbach’s α after item deletion | r | P value |
| --- | --- | --- | --- | --- | --- | --- | --- | --- | --- |
|  | 0 | 1 | 2 | 3 | 4 |  |  |  |  |
| PE1 | 33 | 14 | 69 | 52 | 34 | 0.579 | 0.856 | 0.624 | <0.001 |
| PE2 | 42 | 22 | 59 | 51 | 28 | 0.661 | 0.858 | 0.556 | <0.001 |
| PE3 | 40 | 20 | 55 | 53 | 34 | 0.642 | 0.762 | 0.668 | <0.001 |
| AD1 | 10 | 6 | 35 | 102 | 49 | 0.343 | 0.843 | 0.654 | <0.001 |
| AD2 | 25 | 13 | 36 | 85 | 43 | 0.492 | 0.895 | 0.478 | <0.001 |
| AD3 | 9 | 5 | 33 | 103 | 52 | 0.329 | 0.826 | 0.630 | <0.001 |
| AD4 | 21 | 10 | 35 | 85 | 51 | 0.452 | 0.866 | 0.497 | <0.001 |
| AD5 | 8 | 2 | 33 | 102 | 57 | 0.309 | 0.847 | 0.612 | <0.001 |
| DIS1 | 24 | 28 | 36 | 29 | 85 | 0.553 | 0.947 | 0.612 | <0.001 |
| DIS 2 | 23 | 24 | 36 | 29 | 90 | 0.531 | 0.943 | 0.618 | <0.001 |
| DIS 3 | 19 | 22 | 31 | 37 | 93 | 0.487 | 0.948 | 0.600 | <0.001 |
| DIS4 | 16 | 23 | 30 | 23 | 110 | 0.466 | 0.951 | 0.524 | <0.001 |
| DIS5 | 14 | 17 | 32 | 28 | 111 | 0.429 | 0.956 | 0.514 | <0.001 |
| BC1 | 12 | 16 | 29 | 86 | 59 | 0.399 | 0.951 | 0.686 | <0.001 |
| BC2 | 12 | 11 | 37 | 87 | 55 | 0.387 | 0.968 | 0.704 | <0.001 |
| BC3 | 14 | 12 | 34 | 84 | 58 | 0.405 | 0.964 | 0.701 | <0.001 |
| ET1 | 24 | 16 | 47 | 64 | 51 | 0.510 | 0.974 | 0.769 | <0.001 |
| ET2 | 23 | 15 | 52 | 63 | 49 | 0.503 | 0.978 | 0.776 | <0.001 |
| ET3 | 25 | 15 | 49 | 64 | 49 | 0.515 | 0.979 | 0.779 | <0.001 |
| SE1 | 15 | 10 | 26 | 66 | 85 | 0.401 | 0.986 | 0.652 | <0.001 |
| SE2 | 13 | 9 | 37 | 67 | 76 | 0.390 | 0.912 | 0.666 | <0.001 |
| SE3 | 11 | 9 | 32 | 73 | 77 | 0.371 | 0.914 | 0.657 | <0.001 |
| PC1 | 37 | 15 | 53 | 50 | 47 | 0.613 | 0.901 | 0.768 | <0.001 |
| PC2 | 33 | 12 | 61 | 51 | 45 | 0.540 | 0.963 | 0.838 | <0.001 |
| PC3 | 32 | 13 | 57 | 57 | 43 | 0.536 | 0.939 | 0.823 | <0.001 |

*Note*：PE = Changes in the physical environment; AD = Participatory dialogue (advantages); DIS = Participatory dialogue (disadvantages); BC = Behavioral confidence; ET = Emotional transformation; SE = Changes in the social environment; PC = Practice for change ; CV= Coefficient of variation.

**Table S3.** Results of exploratory factor analysis of the Multi-Theory Model (MTM) of health behavior change scale (*n*=202).

| Dimension | Item | Factor 1 | Factor 2 | Factor 3 | Factor 4 | Factor 5 | Factor 6 |
| --- | --- | --- | --- | --- | --- | --- | --- |
| Changes in the physical environment | PE1 | 0.780 |  |  |  |  |  |
|  | PE2 | 0.814 |  |  |  |  |  |
|  | PE3 | 0.812 |  |  |  |  |  |
| Participatory dialogue (advantages) | AD1 |  | 0.833 |  |  |  |  |
|  | AD2 |  | 0.685 |  |  |  |  |
|  | AD3 |  | 0.869 |  |  |  |  |
|  | AD4 |  | 0.716 |  |  |  |  |
|  | AD5 |  | 0.859 |  |  |  |  |
| Participatory dialogue (disadvantages) | DIS1 |  |  | 0.885 |  |  |  |
|  | DIS 2 |  |  | 0.897 |  |  |  |
|  | DIS 3 |  |  | 0.862 |  |  |  |
|  | DIS4 |  |  | 0.858 |  |  |  |
|  | DIS5 |  |  | 0.855 |  |  |  |
| Behavioral confidence | BC1 |  |  |  | 0.852 |  |  |
|  | BC2 |  |  |  | 0.846 |  |  |
|  | BC3 |  |  |  | 0.858 |  |  |
| Emotional transformation | ET1 |  |  |  |  | 0.844 |  |
|  | ET2 |  |  |  |  | 0.865 |  |
|  | ET3 |  |  |  |  | 0.849 |  |
| Changes in the social environment | SE1 |  |  |  |  |  | 0.882 |
|  | SE2 |  |  |  |  |  | 0.880 |
|  | SE3 |  |  |  |  |  | 0.887 |
| Practice for change | PC1 |  |  |  |  | 0.799 |  |
|  | PC2 |  |  |  |  | 0.849 |  |
|  | PC3 |  |  |  |  | 0.852 |  |

**Table S4.** Cronbach's alpha coefficients and GLB coefficients for each dimension of the extended Multi-Theory Model (MTM) of health behavior change. Scale.

| Dimension | Cronbach's α | GLB |
| --- | --- | --- |
| Changes in the physical environment | 0.844 | 0.890 |
| Participatory dialogue (advantages) | 0.889 | 0.913 |
| Participatory dialogue (disadvantages) | 0.959 | 0.976 |
| Behavioral confidence | 0.976 | 0.976 |
| Emotional transformation | 0.980 | 0.985 |
| Changes in the social environment | 0.928 | 0.931 |

*Note*：GLB = greatest lower bound.

**Figure S1**. Results of validated factor analysis of the extended Multi-Theory Model (MTM) of health behavior change scale (*n*=189).

*Note*: Physical environment = Changes in the physical environment; Social environment = Changes in the social environment; Dialogue (advantages) = Participatory dialogue (advantages); Dialogue (disadvantages) = Participatory dialogue (disadvantages).

**Table S5.** Indicators of fit before and after modification of validated factor analysis models for the extended Multi-Theory Model (MTM) of health behavior change scale (*n*=189).

| Test | Before modification | After modification | Criteria |
| --- | --- | --- | --- |
| *χ*^2^/df | 2.676 | 2.051 | <5 appropriate, <3 good |
| TLI | 0.960 | 0.975 | >0.9 |
| CFI | 0.966 | 0.979 | >0.9 |
| IFI | 0.967 | 0.979 | >0.9 |
| NFI | 0.948 | 0.960 | >0.9 |
| GFI | 0.883 | 0.913 | >0.9 |
| RMSEA | 0.066 | 0.052 | <0.1 appropriate，<0.05 good |

**Table S6**. Path coefficients of the extended Multi-Theory Model (MTM) of health behavior change for self-management behavior in type 2 diabetes mellitus.

| **Pathways** | **β** | **S.E.** | **95%CI** | **P value** |
| --- | --- | --- | --- | --- |
| H1b: Social environment → Dialogue (disadvantages) | 0.20 | 0.08 | (0.04, 0.36) | 0.013 |
| H2b: Physical environment → Dialogue (disadvantages) | -0.00 | 0.08 | (-0.17, 0.16) | 0.987 |
| H1a: Social environment → Dialogue (advantages) | 0.10 | 0.08 | (-0.07, 0.26) | 0.244 |
| H2a: Physical environment → Dialogue (advantages) | -0.01 | 0.08 | (-0.17, 0.16) | 0.914 |
| H3b: Dialogue (disadvantages) → Behavioral confidence | 0.66 | 0.04 | (0.57, 0.74) | <0.001 |
| H3a: Dialogue (advantages) → Behavioral confidence | 0.12 | 0.06 | (0.02, 0.23) | 0.024 |
| H4b: Dialogue (disadvantages) → Emotional transformation | -0.04 | 0.08 | (-0.19, 0.11) | 0.571 |
| H4a: Dialogue (advantages) → Emotional transformation | 0.26 | 0.07 | (0.11, 0.40) | 0.001 |
| H6b: Dialogue (disadvantages) → Diabetes-related skills | -0.02 | 0.08 | (-0.17, 0.13) | 0.812 |
| H6a: Dialogue (advantages) → Diabetes-related skills | 0.03 | 0.08 | (-0.12, 0.18) | 0.707 |
| H7: Behavioral confidence → Self-management | 0.03 | 0.07 | (-0.09, 0.16) | 0.603 |
| H8: Emotional transformation → Self-management | -0.02 | 0.06 | (-0.14, 0.11) | 0.778 |
| H10: Diabetes-related skills → Self-management | 0.16 | 0.07 | (0.03, 0.29) | 0.013 |
| H11a: Dialogue (advantages) → Self-management | -0.27 | 0.27 | (-0.80, 0.26) | 0.316 |
| H11b: Dialogue (disadvantages) → Self-management | 0.04 | 0.22 | (-0.39, 0.47) | 0.872 |
| H12: Social environment → Self-management | 0.23 | 0.08 | (0.07, 0.38) | 0.003 |
| H13: Physical environment → Self-management | 0.25 | 0.07 | (0.10, 0.40) | 0.001 |

*Note:* Hypotheses involving Practice for change (H5a, H5b, and H9) were not tested, as this dimension was excluded during factor analysis due to conceptual overlap with Emotional transformation. Physical environment = Changes in the physical environment; Social environment = Changes in the social environment; Dialogue (Advantages) = Participatory dialogue (advantages); β= Standard coefficients; S.E.= Standard error; 95%CI = 95% Confidence interval.
