# Supplementary material for: Behavioral Mechanisms of a Digital Health Intervention for Self-Management in Type 2 Diabetes Mellitus: Prospective Longitudinal Cohort Study
Source: JMIR Mhealth Uhealth. 2026 Aug 10;14:e79081. doi: 10.2196/79081 (PMC13455579; doi:10.2196/79081)
Supplement: Supplementary file 3 [file mhealth-v14-e79081-s003.docx]

| **Table S1**. Joint Display of Quantitative and Qualitative Findings | | | | | |
| --- | --- | --- | --- | --- | --- |
| **Domain** | **Dimension** | **Quantitative findings** | **Qualitative findings** | **Integration strategy** | **Integrated interpretation** |
| Environmental domain | Changes in the physical environment | Changes in the physical environment showed a direct positive association with diabetes self-management behavior. Physical environment at T0 was directly associated with self-management behavior at T3 (β=0.248, *P*=.001). | The influence of the physical environment was reflected mainly in 2 aspects: (1) the availability and usability of self-management–related resources, such as glucose meters, weighing scales, and exercise aids; and (2) the availability and usability of the PKU Diabetes Butler platform, including blood glucose monitoring, diet and exercise advice, and health education messages. | Confirmation and expansion | Quantitative findings confirmed the direct role of changes in the physical environment in diabetes self-management behavior, while qualitative findings expanded how this operated in practice through access to self-management resources and digital platform functions. |
| Environmental domain | Changes in the social environment | Changes in the social environment showed a direct positive association with diabetes self-management behavior. Social environment at T0 was directly associated with self-management behavior at T3 (β=0.226, *P*=.003). In addition, social environment at T0 was associated with participatory dialogue disadvantages at T1 (β=0.203, *P*=.013), suggesting links with subsequent cognitive and attitudinal processes. | The influence of the social environment was reflected mainly in 3 aspects: (1) family support and supervision, including reinforcement of behavioral confidence and assistance with self-management; (2) peer or mutual-support networks, together with support from the PKU Butler platform, such as practical advice and experience sharing; and (3) broader supportive social contexts, including community health services and insurance-related support. | Confirmation and expansion | Quantitative findings confirmed the direct role of changes in the social environment in diabetes self-management behavior, while qualitative findings expanded its meaning by showing that social support could both directly facilitate self-management and shape subsequent cognitive and attitudinal processes. |
| Cognitive domain | Participatory dialogue (advantages and disadvantages) | In the final quantitative model, participatory dialogue did not show a direct significant association with self-management behavior, but was associated with subsequent attitudinal and skills-related processes. Participatory dialogue advantages at T1 were associated with behavioral confidence at T2 (β=0.123, *P*=.024) and emotional transformation at T2 (β=0.257, *P*=.001). Participatory dialogue disadvantages at T1 were associated with behavioral confidence at T2 (β=0.655, *P*<.001). Because participatory dialogue disadvantages were reverse-coded, higher scores indicated fewer perceived disadvantages. | Participatory dialogue influenced self-management mainly through 3 forms of interaction: (1) two-way communication with the PKU Diabetes Butler platform, through which participants obtained detailed and practical diabetes management information; (2) interaction with health care professionals, including adjustment of diet and physical activity based on clinical advice; and (3) interaction with other people, including reflection prompted by peers or major health-related events. | Expansion | Quantitative findings suggested that participatory dialogue was linked primarily to subsequent attitudinal and skills-related processes rather than directly to self-management behavior. Qualitative findings further expanded its mechanism by showing how human-computer interaction, patient-provider interaction, and broader social interaction supported self-management. |

| Attitudinal and skills domain | Behavioral confidence | Behavioral confidence was retained in the final quantitative model, but no significant direct pathway to self-management behavior was observed. | Behavioral confidence was reflected mainly in 2 aspects: (1) confidence in one’s own ability to manage diabetes and maintain glycemic control; and (2) trust in the PKU Diabetes Butler platform, including confidence in the health information provided and in the underlying AI technology. | Expansion | Quantitative findings did not support a significant direct effect of behavioral confidence on self-management behavior, whereas qualitative findings expanded its meaning by showing that behavioral confidence was expressed through both self-efficacy and trust in the digital platform. |
| --- | --- | --- | --- | --- | --- |
| Attitudinal and skills domain | Emotional transformation | Emotional transformation was retained in the final quantitative model, but no significant direct pathway to self-management behavior was observed. | Emotional transformation was reflected mainly in 2 aspects: (1) improved emotional regulation, such as reduced anxiety and better adjustment of mood through platform use; and (2) transforming negative emotions into motivation for self-management. | Expansion | Quantitative findings did not support a significant direct effect of emotional transformation on self-management behavior, whereas qualitative findings expanded its role as a process through which participants converted negative emotions into motivation for sustained self-management. |
| Attitudinal and skills domain | Practice for change | Practice for change was initially included during scale development but was not retained as an independent factor in the final quantitative model. | Practice for change was reflected mainly in 2 aspects: (1) behavioral recording and reflection, such as reminders, self-monitoring, and reviewing pushed messages; and (2) behavioral adjustment and maintenance, including modifying glucose monitoring frequency, dietary habits, and other self-management behaviors over time. | Expansion | Practice for change was not retained as an independent quantitative factor, but qualitative findings clearly showed its importance in behavioral maintenance through reminders, monitoring, reflection, and ongoing adjustment. |
| Attitudinal and skills domain | Diabetes-related skills | Diabetes-related skills showed a direct positive association with diabetes self-management behavior. Diabetes-related skills at T2 were directly associated with self-management behavior at T3 (β=0.162, *P*=.013). | The influence of diabetes-related skills was reflected mainly in 4 aspects: (1) blood glucose monitoring skills; (2) dietary management skills; (3) physical activity management skills; and (4) medication management skills. | Confirmation and expansion | Quantitative findings confirmed the direct role of diabetes-related skills in diabetes self-management behavior, while qualitative findings expanded the specific content of these skills across glucose monitoring, diet, exercise, and medication management. |
