# Supplementary material for: Behavioral Mechanisms of a Digital Health Intervention for Self-Management in Type 2 Diabetes Mellitus: Prospective Longitudinal Cohort Study
Source: JMIR Mhealth Uhealth. 2026 Aug 10;14:e79081. doi: 10.2196/79081 (PMC13455579; doi:10.2196/79081)
Supplement: Supplementary file 4 [file mhealth-v14-e79081-s004.docx]

**Good Reporting of A Mixed Methods Study (GRAMMS)**

| **Guideline** | **Section: page** |
| --- | --- |
| Describe the justification for using a mixed methods approach to the research question | Study Objective; Study Design  pp 3–4 |
| Describe the design in terms of the purpose, priority and sequence of methods | Study Design; Statistical Analyses  pp 3–5 |
| Describe each method in terms of sampling, data collection and analysis | Setting and Participants; Quantitative Data Collection; Qualitative Data Collection; Measurements; Statistical Analyses  pp 3–5 |
| Describe where integration has occurred, how it has occurred and who has participated in it | Statistical Analyses (joint display integration); Integration of Quantitative and Qualitative Data  pp 5, 10–11 |
| Describe any limitation of one method associated with the present of the other method | Discussion; Limitations  pp 11–14 |
| Describe any insights gained from mixing or integrating methods | Integration of Quantitative and Qualitative Data; Discussion  pp 10–14 |

## **References**

1. O'Cathain A, Murphy E, Nicholl J. The quality of mixed methods studies in health services research. J Health Serv Res Policy. 2008;13(2):92-98.
